# Massive surge of mRNA expression of clonal B-cell receptor in patients with COVID-19

**DOI:** 10.1016/j.heliyon.2021.e07748

**Published:** 2021-08-10

**Authors:** Yohei Funakoshi, Goh Ohji, Kimikazu Yakushijin, Kei Ebisawa, Yu Arakawa, Jun Saegusa, Hisayuki Matsumoto, Takamitsu Imanishi, Eriko Fukuda, Takaji Matsutani, Yasuko Mori, Kentaro Iwata, Hironobu Minami

**Affiliations:** a Division of Medical Oncology/Hematology, Department of Medicine, Kobe University Hospital and Graduate School of Medicine, Kobe, Japan; b Division of Infection Disease Therapeutics, Department of Microbiology and Infectious Diseases, Kobe University Graduate School of Medicine, Kobe, Japan; c Department of Clinical Laboratory, Kobe University Hospital, Kobe, Japan; d National Institute of Advanced Industrial Science and Technology, Cellular and Molecular Biotechnology Research Institute, Tokyo, Japan; e Research & Development Department, Repertoire Genesis Inc., Ibaraki, Japan; f Division of Clinical Virology, Center for Infectious Diseases, Kobe University Graduate School of Medicine, Kobe, Japan; g Cancer Center, Kobe University Hospital, Kobe, Japan

**Keywords:** Coronavirus disease 2019, Severe acute respiratory syndrome coronavirus 2, B-cell receptor, Repertoire assay

## Abstract

**Background:**

Antibody production is one of the primary mechanisms for recovery from coronavirus disease 2019 (COVID-19). It is speculated that massive clonal expansion of B cells, which can produce clinically meaningful neutralizing antibodies, occurs in patients who recover on the timing of acquiring adaptive immunity.

**Methods:**

To evaluate fluctuations in clonal B cells and the size of the clones, we chronologically assessed the B-cell receptor (BCR) repertoire in three patients with COVID-19 who recovered around 10 days after symptom onset.

**Results:**

We focused on the three dominant clonotypes (top 3) in each individual. The percentage frequencies of the top 3 clonotypes increased rapidly and accounted for 27.8 % on day 9 in patient 1, 10.4 % on day 12 in patient 2, and 10.8 % on day 11 in patient 3, respectively. The frequencies of these top 3 clonotypes rapidly decreased as the patients’ clinical symptoms improved. Furthermore, BCR network analysis revealed that accumulation of clusters composed of similar complementarity-determining region 3 (CDR3) sequences were rapidly formed, grew, and reached their maximum size around 10 days after symptom onset.

**Conclusions:**

BCR repertoire analysis revealed that a massive surge of some unique BCRs occurs during the acquisition of adaptive immunity and recovery. The peaks were more prominent than expected. These results provide insight into the important role of BCRs in the recovery from COVID-19 and raise the possibility of developing neutralizing antibodies as COVID-19 immunotherapy.

## 1 Introduction

Adaptive immunity has been identified as one of the primary mechanisms for protecting humans from infectious diseases [1]. Specifically, antibodies produced by B cells can effectively protect against certain pathogens through neutralization or other antibody-mediated immune mechanisms [1]. During the early phase of infection, B-cell activation is triggered by the binding of foreign antigens to the B-cell receptor (BCR) [2]. Maintenance of sustained BCR signaling by the antigen leads to clonal expansion, class-switch recombination, and affinity maturation, resulting in abundant production of specific antibodies [2, 3, 4].

To react with a large variety of antigens, diverse BCRs are generated by the mechanism of genetic rearrangement (known as V (D) J gene recombination) and somatic hypermutation [5, 6]. Specifically, complementarity-determining region 3 (CDR3), which is encoded by the V-J or V-D-J junctions in BCR, is the most hypervariable region and most important structure in antigen recognition, thereby determining the fates of the developing and responding cells [6]. Recent developments of BCR repertoire assays, which can be used to comprehensively analyze the number of reads with a unique combination of V-J genes and CDR3 amino acids, has enabled studies of the convergence and diversification of BCR. The BCR repertoire assay has been used for immunological research in a variety of fields, including infectious disease, autoimmune disease, and cancer.

Coronavirus disease 2019 (COVID-19), caused by severe acute respiratory syndrome coronavirus 2 (SARS-CoV-2), rapidly became a pandemic after it was discovered in December 2019 [7]. The pandemic has had a major public health impact. Although the pathophysiology and recovery mechanisms of COVID-19 remain poorly understood and there is no standard treatment, adaptive immunity, including antibody neutralization, is thought to play an important role in recovery from COVID-19 [7]. It has been reported that SARS-CoV-2-specific antibodies develop 10–15 days after the onset of symptoms [7, 8]. In practice, we experienced recovered cases associated with the emergence of SARS-CoV-2 specific antibodies around 10 days after symptom onset.

Based on this clinical finding, we hypothesized that massive clonal expansion of B cells, which can produce clinically meaningful neutralizing antibodies, occurs in patients who recover approximately 10 days after onset. The relationship between the clinical course and fluctuation of B-cell clones in patients with COVID-19 over time has not been studied previously. To determine the fluctuation and magnitude of these clonal B cells, we chronologically assessed expansion of the BCR repertoire in patients with COVID-19 who recovered approximately 10 days after onset. Identification of expanding clones related to recovery is useful for revealing the recovery mechanism of COVID-19 and developing effective antibody therapy. The aim of this study was to clarify the kinetics of the BCR repertoire to provide a foundation for further studies of COVID-19.

## 2 Material and methods

### 2.1 Patients

Peripheral blood samples were collected from 45 patients with COVID-19 at Kobe University Hospital between August 2020 and December 2020. Among them, three patients showing fever resolution at around 10 days after onset were selected to analyze the BCR repertoire. All patients were classified as having mild illness and were not administrated immunosuppressive drugs such as dexamethasone. COVID-19 was suspected based on flu-like symptoms and confirmed by a quantitative reverse transcription-polymerase chain reaction assay for SARS-CoV-2. The study protocol was approved by Kobe University Hospital Ethics Committee (No. B2056704). This study was conducted in accordance with the principles of the Declaration of Helsinki. The patients provided written informed consent for this research.

### 2.2 Sample collection and processing

Blood samples were collected up to 5 times over 4 weeks. Heparin-containing tubes were used to collect peripheral blood samples from patients with COVID-19. Peripheral blood mononuclear cells (PBMCs) were isolated from the blood by density gradient centrifugation using Ficoll-Paque Plus (GE Healthcare, Little Chalfont, UK) and SepMate-50 tubes (STEMCELL Technologies, Vancouver, Canada). PBMC isolation was performed according to the manufacturer's instructions of SepMate-50 tubes (STEMCELL Technologies). PBMC samples were stored with CELLBANKER (ZENOGEN PHARMA, Fukushima, Japan) in a freezer at -80 °C. TRIzol LS (Thermo Fisher Scientific, Waltham, MA, USA) was used to extract total RNA and SARS-CoV-2 inactivation. Total RNA was extracted and purified with an RNeasy Mini Kit (Qiagen, Hiden, Germany) according to the manufacturer's instructions. The RNA amounts and purity were measured with an Agilent 2200 TapeStation (Agilent Technologies, Santa Clara, CA, USA).

### 2.3 SARS-CoV-2 specific immunoglobulin G/M antibody assay and neutralization test

SARS-CoV-2 specific immunoglobulin G (IgG) and IgM were examined using an immunochromatographic test kit (2019-nCoV Ab Test; INNOVITA, Hebei, China) as previously described [9].

To evaluate the neutralizing activity of the serum, we performed a neutralization test against 102 SARS-CoV-2 (Biken-2 strain), which was provided by the Research Foundation for Microbial Diseases of Osaka University (BIKEN). For this purpose, 4 × 10^4^ Vero E6 (TMPRSS2) cells [10] per well were seeded into 96-well tissue culture microplates at 24 h before the assay. Duplicate samples of a three-fold serial dilution of heat-inactivated (56 °C, 30 min) patient serum were prepared using Dulbecco's Modified Eagle's Medium as the diluent and mixed with 100 tissue culture infectious dose 50 of virus and incubated at 37 °C for 1 h. After incubation, the serum-virus mixture was added to Vero E6 (TMPRSS2) cells and incubated at 37 °C for 3 days. The neutralizing antibody titer was determined as the highest serum dilution that did not exhibit a cytopathic effect when observed under a microscope. These experiments were repeated three times independently.

### 2.4 B-cell receptor repertoire analysis

BCR repertoire analysis was performed using unbiased next-generation sequencing developed by Repertoire Genesis, Inc. (Osaka, Japan) [11]. Briefly, cDNA was synthesized from total RNA using the polyT18 primer (BSL-18E) and Superscript III reverse transcriptase (Invitrogen, Carlsbad, CA, USA). After synthesizing double-strand (ds)-cDNA, the P10EA/P20EA dsDNA adaptor was ligated and cut with the *Not*I restriction enzyme. Nested PCR was performed with KAPA HiFi DNA Polymerase (Kapa Biosystems, Woburn, MA, USA) using IgG constant region-specific primers (CG1 and CG2) and P20EA. The amplicon library was prepared by amplification of the second PCR products using P22EA-ST1 and CG-ST1-R. Index (barcode) sequences were added by amplification with the Nextera XT Index Kit v2 Set A (Illumina, San Diego, CA, USA). Sequencing was performed using the Illumina MiSeq paired-end platform (2 × 300 bp). BCR sequences were assigned based on identity with reference sequences from the international ImMunoGeneTics information system® (IMGT) database (http://www.imgt.org) using repertoire analysis software originally developed by Repertoire Genesis, Inc. (Osaka, Japan).

### 2.5 Calculation of metrics and network analysis

Data analysis was performed and graphics were prepared using the packages implemented in R software (version 4.0.2). The Shannon-Weaver diversity index and Levenshtein distance (edit distance) were calculated using the vegan 2.5–7 package. Network analyses were performed using igraph 1.2.6 (https://igraph.org/r/) implemented in R. The most 1,000 frequent BCR clonotypes (nodes) were connected by edges defined by a Levenshtein distance of less than or one amino acid sequence of CDR3. The network was created from adjacency matrices constructed using the graph.adjacency (mode = “undirected”) function of igraph, and the community (cluster) was extracted using the fastgreedy.community function. Graphics were drawn using ggplot2 version 3.3.2.

## 3 Results

### 3.1 Patient characteristics

**Figure 1:**
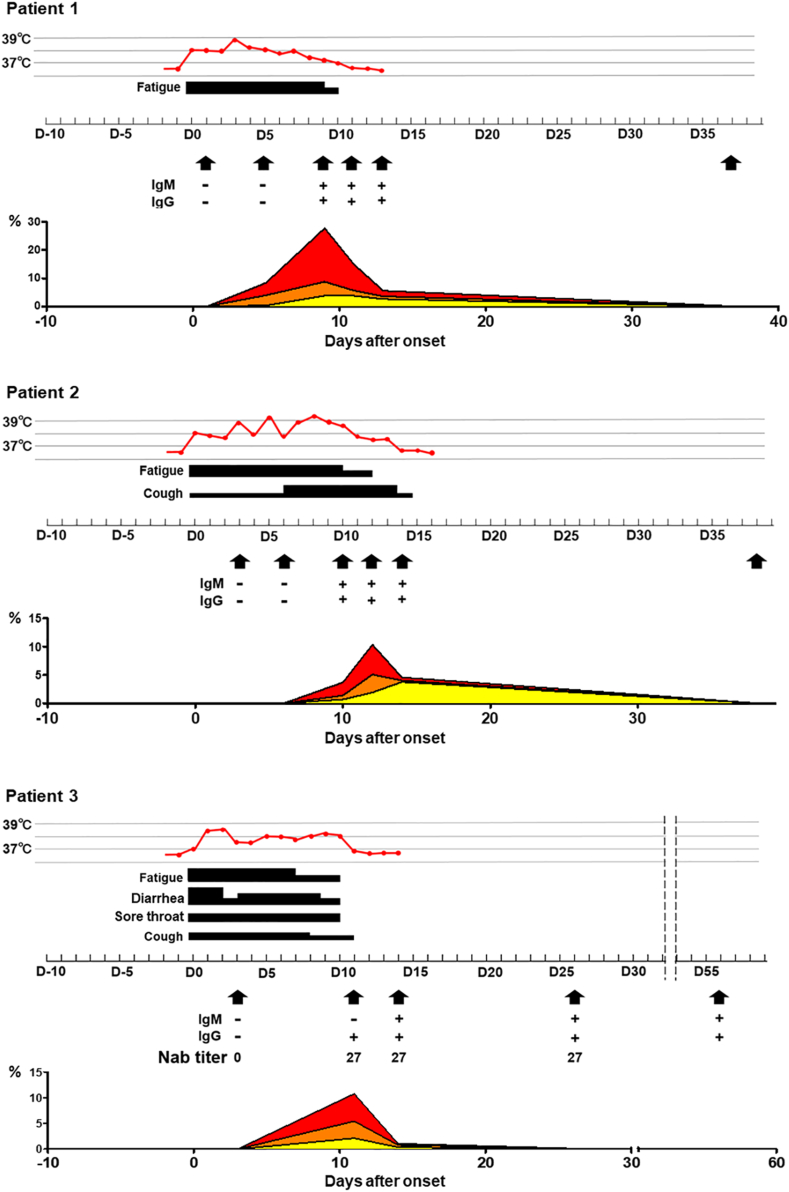
Clinical course of the three patients with COVID-19. The red line represents body temperature. The black box shows the symptoms of COVID-19 (fatigue and cough). Days (D) indicate the day after symptom onset. The day of sample collection is indicated by arrows. SARS-CoV-2 antibody positivity is shown by positive (+) or negative (−) at the day of sample collection. The percentage frequencies of the top 1 (red), top 2 (orange), and top 3 (yellow) BCR clonotypes are shown by stacked area charts.

Three patients with fever resolution around 10 days after onset were selected for this study (Figure 1). The patient characteristics are shown in Table 1. None of the patients had any underlying autoimmune or autoinflammatory diseases and was taking any medications that affect systemic immunity. None of the patients had been administered any immunosuppressive agents, including dexamethasone, to treat COVID-19. All patients were Japanese.

**Table 1:** Patient characteristics.

	Patient 1	Patient 2	Patient 3
Age (years)	60	44	36
Sex	Female	Male	Male
Symptoms other than fever	Fatigue	Fatigue, cough	Fatigue, diarrhea, sore throat, cough
Medications for COVID-19	Acetaminophen	Acetaminophen, dextromethorphan	Acetaminophen, dextromethorphan, codeine phosphate
Complications	Chronic obstructive pulmonary disease, myocardial infarction, asthma	Hypertension, dyslipidemia	Nonalcoholic steato-hepatitis
Commonly used medications	Warfarin, bisoprolol, spironolactone, losartan, pranlukast, omeprazole, budesonide/formoterol (inhalation)	Olmesartan	

### 3.2 SARS-CoV-2-specific IgG and IgM antibodies

To evaluate antibody production in these patients, five or six blood samples were obtained from the patients. Over the clinical course of their illness, all three patients seroconverted and developed IgG and IgM antibodies specific for SARS-CoV-2 (Figure 1). Seroconversion of anti-SARS-CoV-2 IgM antibodies was observed on days 9, 10, and 14 in patients 1, 2, and 3, respectively. Simultaneously, IgG antibodies were detected on days 9, 10, and 11 in patients 1, 2, and 3, respectively, and the samples were continuously positive for antibodies even after the improvement of clinical symptoms. We could perform a neutralization assay on a blood sample from patient 3, and detected neutralization activity from day 11 after onset (Figure 1).

### 3.3 Fluctuation of B-cell receptor repertoire after SARS-CoV-2 infection

**Figure 2:**
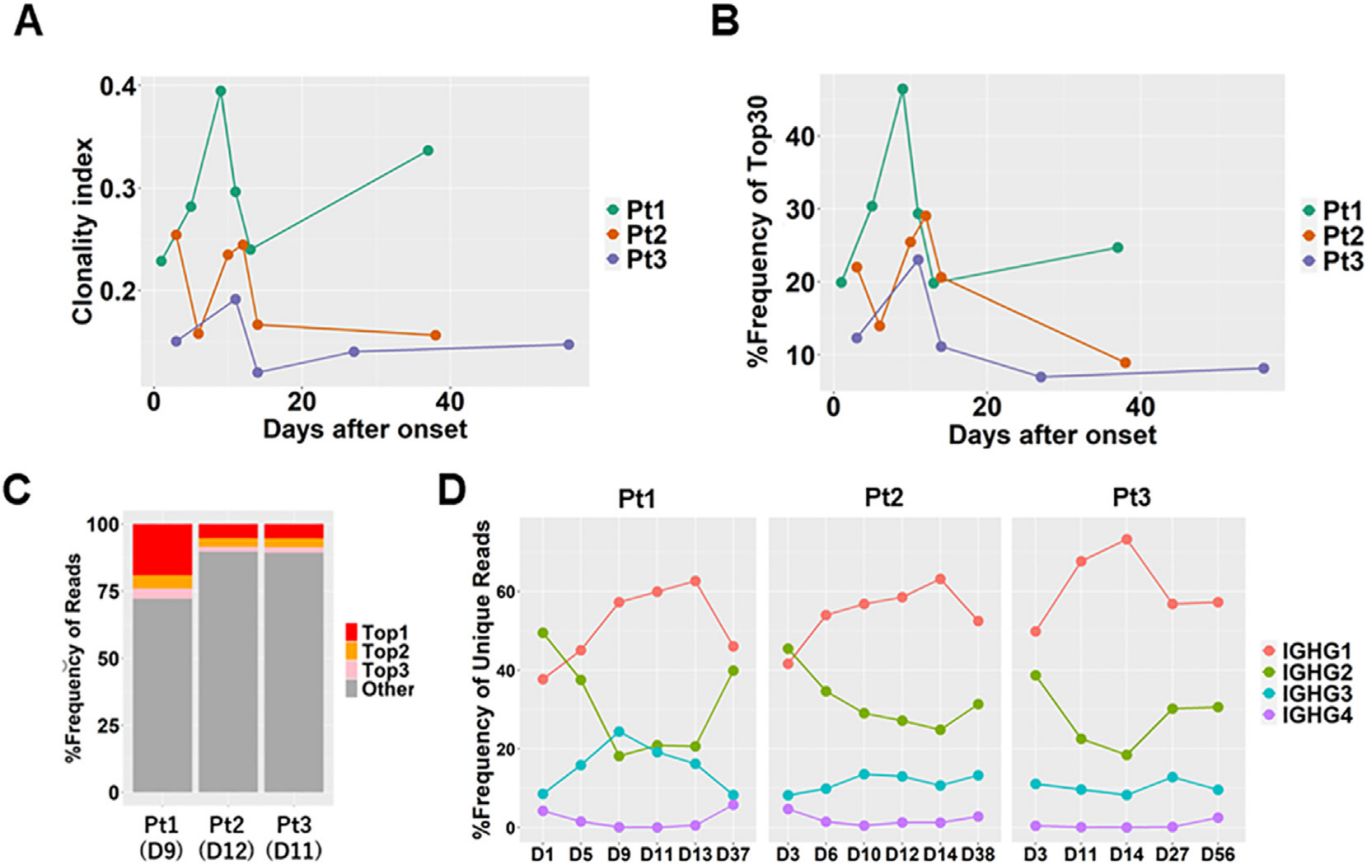
(A) Change in B-cell receptor (BCR) clonality in three patients after SARS-CoV-2 infection. Clonality index is calculated as 1 – normalized Shannon-Weaver index. (B) Change in occupancy of the 30 most frequent BCR clonotypes in three patients after SARS-CoV-2 infection (B). Percentage of the number of reads of the top 30 clonotypes as a proportion of the total number of reads is plotted against days after onset. (C) Occupancy of the top 1, top 2, and top 3 clonotypes at the peak of fluctuation. The percentage frequencies of the top 1 (red), top 2 (orange), top 3 (pink) most common, and other clonotypes (gray) on day 9 in patient 1, day 12 in patient 2, and day 11 in patient 3, are shown in the stacked bar plot. (D) Change in frequencies of IgG subclasses in three patients after SARS-CoV-2 infection. The percentage frequencies of unique reads bearing IGHG1 (IgG1, orange), IGHG2 (IgG2, green), IGHG3 (IgG3, blue) and IGHG4 (IgG4, purple) are plotted against days since onset.

To assess alterations in the BCR repertoire after SARS-CoV-2 infection, high-throughput sequencing of IgG transcripts was performed with PBMCs collected from three patients at the time of serum collection (Figure 1). A total of 4,768,753 reads and 464,981 unique reads were obtained from 17 samples (mean: 280,512 and 27,352, respectively). The unique IgG reads assigned by the IMGT reference sequences were ranked according to their abundance. We focused on the three dominant clonotypes (top 3) in each patients' peak (Figure 1 and Table 2). The percentage frequencies of the top 3 clonotypes increased rapidly and accounted for 27.8 % on day 9, 10.4 % on day 12, and 10.8 % on day 11 in patients 1, 2, and 3, respectively (Figures 1, 2C and Table 2). The frequencies of these top 3 clonotypes rapidly decreased as the patients’ clinical symptoms improved and were almost undetectable at the time of the last sampling on days 37–56 (Figure 1). Fluctuations in these top 3 clonotypes with clinical manifestations are shown in Figure 1.

**Table 2:** Percentage of the top 3 clonotypes in each patient.

Patient and day	Rank	IGHV	IGHD	IGHJ	IGHC	CDR3	%
Patient 1, Day 9	1	IGHV4-34	IGHD3-22	IGHJ6	IGHG3	CARGKSENIMVVVVITGYYYYMDVW	19.08
	2	IGHV4-61	IGHD3-3	IGHJ6	IGHG1	CAREEFLEWLFPPLYYYNGMDVW	4.95
	3	IGHV2-5	—	IGHJ4	IGHG1	CTHKPPNIGFDLWFDYW	3.79
Patient 2, Day 12	1	IGHV3-33	IGHD5-12	IGHJ6	IGHG1	CVRVRYRGYDYSLFYYDMDVW	5.27
	2	IGHV3-9	IGHD2-21	IGHJ4	IGHG1	CAKAQGGLVVVTGGNFFDHW	3.17
	3	IGHV3-74	—	IGHJ4	IGHG2	CTRGDSNGSPDYW	1.92
Patient 3, Day 11	1	IGHV4-34	—	IGHJ6	IGHG3	CARGVGVPGIFYHTFYYQGLDVW	5.32
	2	IGHV1-8	—	IGHJ4	IGHG2	CARDGPDSGDIHFW	3.31
	3	IGHV2-70D, IGHV2-70	—	IGHJ2	IGHG1	CARTAVGGTSWHFDLW	2.13

CDR3, complementarity-determining region 3; IGH, immunoglobulin heavy chain; IGHV, IGH variable; IGHJ, IGH joining; IGHD, IGH diversity.

### 3.4 Longitudinal changes of B-cell receptor diversity

The Shannon-Weaver diversity index of the BCR repertoire was calculated for longitudinal samples to clarify changes in the overall repertoire at post-infection follow-up. The peaks of the clonality index calculated as 1 – normalized Shannon-Weaver index rapidly were observed around day 10 (Figure 2A). Similarly, the percentage occupancy of the 30 most dominant BCR clonotypes increased at around day 10 and decreased thereafter (Figure 2B). These changes coincided with the increase in the top 3 clonotypes (Figures 1, 2B). Furthermore, the frequencies of BCR clonotypes with IGHG1 constant sequence (IgG1) increased after infection relative to IgG2 in all patients (Figure 2D). These results indicate that the alteration in the BCR repertoire immediately after SARS-CoV-2 infection was caused by clonal expansion of several BCR clonotypes.

### 3.5 Cluster formation of B-cell receptor clonotypes

**Figure 3:**
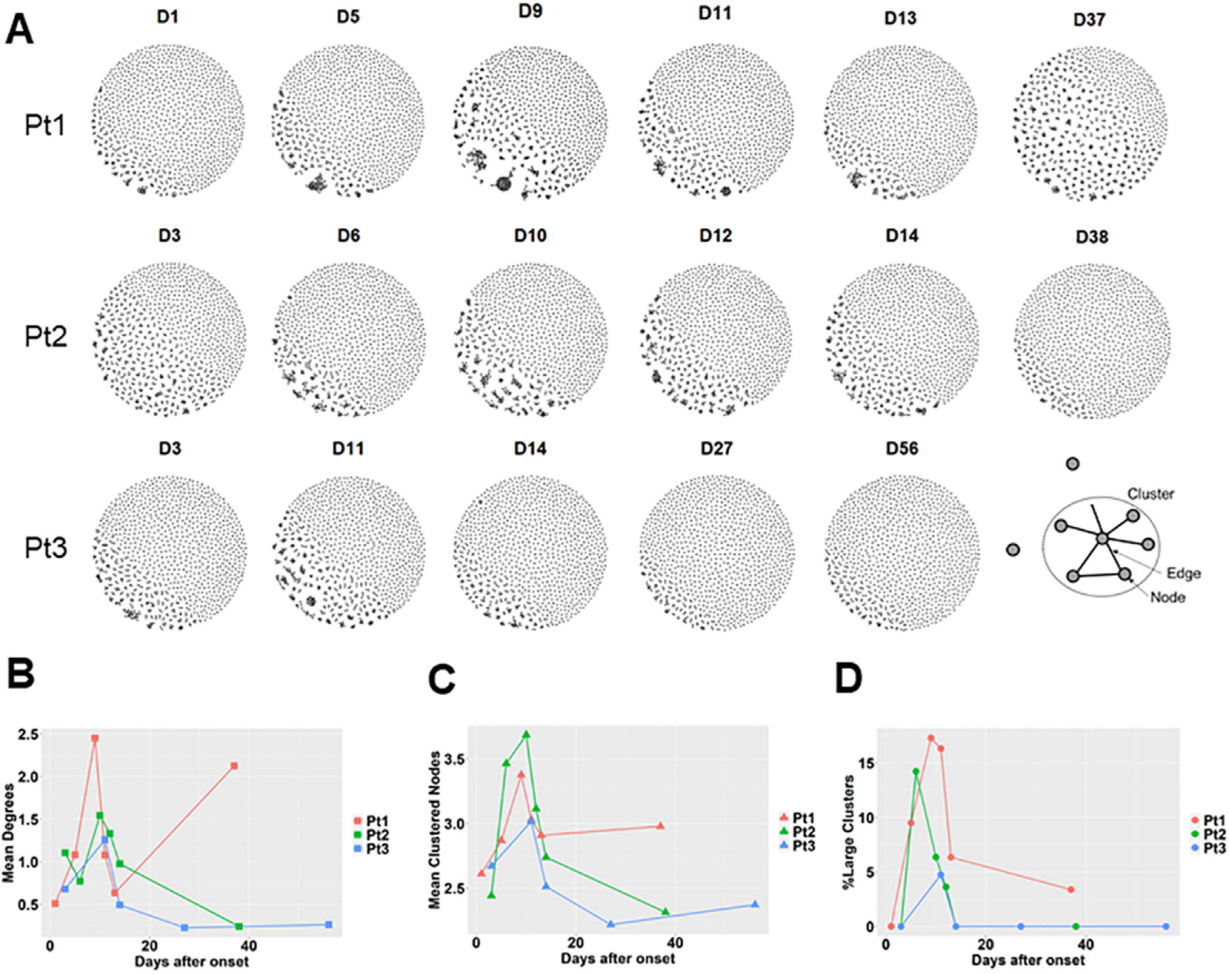
(A) Networks formed by unique B-cell receptor (BCR) clonotypes after SARS-CoV-2 infection. Each node represents a single BCR clonotype with an identical IGHV, IGHJ, IGHC and complementarity-determining region 3 (CDR3) sequence. The node was connected by edges defined by ≤ 1 Levenshtein distance in CDR3 sequence. Node size is the percentage frequency of each BCR clonotype. (B) Change of degree in the BCR network after SARS-CoV-2 infection. Mean number of degrees (number of edges directly bound to each node) in the node is plotted against days after onset. (C) Change in number of clustered nodes after SARS-CoV-2 infection. The mean number of nodes in each cluster is plotted against days after onset. (D) Change in occupancy of larger clusters after SARS-CoV-2 infection. The percentage frequencies of larger clusters formed by more than ten nodes are plotted against days after onset.

Analysis of networks formed by BCR clonotypes revealed convergence and diversification of the BCR repertoire and evolutionary relationships among closely related sequences. To evaluate the evolutionary relationships among BCR clonotypes after SARS-CoV-2 infection, a network was constructed based on differences in the amino acid sequence of CDR3 between unique BCR clonotypes (Figure 3A). Each node represents a single BCR clonotype with identical IGHV, IGHJ, IGHC, and CDR3 sequences, and nodes were connected to similar BCR clonotypes with no or one amino acid difference in the CDR3 region (Levenshtein distance = 1) (Figure 3A). Groups interconnected by a large number of nodes formed clusters. The BCR clusters were rapidly formed, grew, and reached their maximum size at around 10 days after symptom onset. Subsequently, the clusters rapidly disappeared. The mean number of degrees (number of directly connected nodes) peaked on around day 10 (Figure 3B), indicating that the BCR clonotype, in which CDR3 sequences were more similar to each other, had emerged by around day 10 after onset. Similarly, the mean number of nodes forming a BCR cluster increased on around day 10 (Figure 3C). The percentage occupancy of large clusters formed by more than ten nodes reached a maximum at approximately 10 days of 17.3 %, 14.2 %, and 4.7 % in patients 1, 2, and 3, respectively (Figure 3D).

## 4 Discussion

In this study, we detected a massive surge of the mRNA expression of some unique BCR clonotypes in patients after SARS-CoV-2 infection. The timing of the peak expression of clonotypes coincided with the emergence of SARS-CoV-2 specific antibodies, reflecting the time of acquiring adaptive immunity. Appearance of the peak also coincided with the onset of the patients’ clinical recovery. These fluctuating B-cell clones may play a key role in the production of clinically effective neutralizing antibodies. The peaks were more prominent than expected. Although the surge lasted for only a few days, the activation was substantial. Therefore, these B cells may be obtained by single-cell analysis during this phase. BCR information from these B cells may contribute to the development of neutralizing antibodies, which lead to clinical recovery.

The usefulness of BCR network analysis has been reported in patients with chronic lymphocytic leukemia [12] and in B-cell populations from mice and humans [13]. In this study, network analysis revealed that SARS-CoV-2 infection increased the number and size of clusters formed by BCR clonotypes. Accumulation of large clusters composed of similar CDR3 sequences may show that diversification of antibodies by frequent somatic hypermutation and subsequent selection of high-affinity antibodies peaked at around 10 days after symptom onset, leading to affinity maturation. Dominant BCR clonotypes, such as the top 1 clonotype of patient 1, also formed a large cluster of BCR clonotypes containing identical CDR3 with different IgG subclasses. This suggests that although the antigen specificities are unknown, dominant BCR clonotypes induced by infection are under diversification and selection pressure and undergo class-switch recombination.

Notably, our results revealed an increase in the frequency of IgG1 unique clonotypes. IgG1 and IgG3 are reportedly the dominant IgG subclasses produced in response to SARS-CoV-2 infection [14]. This suggests that Th1 cytokines (such as interferon-γ and interleukin-2) induced by infection contributed to the shift from the IgG2 to IgG1 subclasses.

In this study, the expanded clones and clusters were not maintained and rapidly decreased within a few days. Based on the clinical course, we predicted that activated BCR signaling also decreased as the viral load decreased. This is consistent with previous research showing that a very limited number of cells among expanded cells differentiate into long-lived memory B cells, with most cells rapidly disappearing. This is in contrast to antibodies with long half-lives, which accumulate over a long period. Although some research groups have reported on the BCR repertoire, to our knowledge, there have been no similar studies focusing on fluctuations in the BCR repertoire over a very short period [15, 16, 17, 18, 19].

Our study had some limitations. First, samples from only three patients were analyzed. To confirm the clinical significance of the observed changes, further investigation of a larger number of patients is needed. Additionally, we did not perform this assay in patients who rapidly recovered with fever resolution in only a few days and long-term non-recovered patients. A much larger number of cases and comparison of patients with various clinical courses must be evaluated. Additionally, comparison with other chronic viral infections is required. Second, we did not confirm whether antibodies containing these unique sequences connect to SARS-CoV-2 as the antigen and possess neutralization activity. Although we focused only on sequences of IgG heavy-chains, we are working on developing a method to identify sequences of both heavy- and light-IgG chains by single-cell analysis and an antibody for identifying antigens and estimating neutralization activity. Third, recent studies reported a relationship between the T cell receptor repertoire and recovery from COVID-19 [20, 21]; therefore, further studies should focus on not only the BCR but also the T cell receptor repertoire.

In conclusion, chronological BCR repertoire analysis revealed that a massive surge of the mRNA expression of some unique BCRs occurs during the acquisition of adaptive immunity and recovery. These results may contribute to the development of neutralizing antibodies as COVID-19 therapy.

## Declarations

### Author contribution statement

Yohei Funakoshi: Conceived and designed the experiments; Analyzed and interpreted the data; Contributed reagents, materials, analysis tools or data; Wrote the paper.

Goh Ohji, Kimikazu Yakushijin: Conceived and designed the experiments; Analyzed and interpreted the data; Contributed reagents, materials, analysis tools or data.

Kei Ebisawa, Yu Arakawa: Contributed reagents, materials, analysis tools or data.

Jun Saegusa, Hisayuki Matsumoto, Takamitsu Imanishi: Performed the experiments; Contributed reagents, materials, analysis tools or data.

Eriko Fukuda: Analyzed and interpreted the data; Contributed reagents, materials, analysis tools or data.

Takaji Matsutani: Performed the experiments; Analyzed and interpreted the data; Contributed reagents, materials, analysis tools or data.

Yasuko Mori, Hironobu Minami: Conceived and designed the experiments; Analyzed and interpreted the data.

Kentaro Iwata: Conceived and designed the experiments; Contributed reagents, materials, analysis tools or data.

### Funding statement

This work was supported by the 10.13039/100009619Japan Agency for Medical Research and Development (AMED) [grant number 20he0522002] and Post Corona Hyogo Prefecture Subsidized Project.

### Data availability statement

Data will be made available on request.

### Declaration of interests statement

The authors declare the following conflict of interests: Takaji Matsutani is an employee of Repertoire Genesis Inc..

### Additional information

No additional information is available for this paper.
